# Blockchain Mechanism and Symmetric Encryption in A Wireless Sensor Network

**DOI:** 10.3390/s20102798

**Published:** 2020-05-14

**Authors:** Alma E. Guerrero-Sanchez, Edgar A. Rivas-Araiza, Jose Luis Gonzalez-Cordoba, Manuel Toledano-Ayala, Andras Takacs

**Affiliations:** Faculty of Engineering, Autonomous University of Queretaro, Querétaro 76017, Mexico; aguerrero69@alumnos.uaq.mx (A.E.G.-S.); erivas@uaq.mx (E.A.R.-A.); jose.gonzalez.cordoba@uaq.mx (J.L.G.-C.); toledano@uaq.mx (M.T.-A.)

**Keywords:** Internet of Things (IoT), Wireless Sensor Network (WSN), blockchain, Advanced Encryption Standard (AES), decentralized network

## Abstract

The Internet of Things (IoT) paradigm allows the connection and exchange of information between millions of smart devices. This paradigm grows and develops exponentially as do the risks and attacks on IoT infrastructures. Security, privacy, reliability, and autonomy are the most important requirements in IoT Systems. If these issues are not guaranteed, the IoT system could be susceptible to malicious users and malicious use. In centralized IoT systems, attacks and risks are greater, especially when data is transmitted between devices and shared with other organizations. To avoid these types of situations, this work presents a decentralized system that guarantees the autonomy and security of an IoT system. The proposed methodology helps to protect data integrity and availability based on the security advantages provided by blockchain and the use of cryptographic tools. The accuracy of the proposed methodology was measured on a temperature and humidity sensing IoT-based Wireless Sensor Network (WSN). The obtained results prove that the proposal fulfils the main requirements of an IoT system. It is autonomous, secure to share and send information between devices and users, has privacy, it is reliable, and the information is available in the infrastructure. Furthermore, this research demonstrates that the proposal is less susceptible to the most frequent attacks against IoT systems, such as linking attack, man in the middle, and Distributed Denial of Service (DDoS) attack.

## 1. Introduction

The Internet of Things (IoT) can be seen as a network of physical devices, with the ability to collect and share different types of information in any location, time, medium, and context [1]. With these characteristics, IoT can be applied in many different fields, for example, in health care, monitoring, and sending vital information about the patients to the doctors to make their diagnosis [2]. In the military, the IoT technology is used for secure information exchange between remote units with the use of low-consumption wireless sensor networks [3]. Likewise, IoT plays a vital role in smart cities, buildings, and homes, with various contributions to improve the quality of life of the inhabitants. The authors of Reference [4] implemented an IoT video surveillance system with smart cameras using facial recognition to increase home security. Although data security and autonomy have great importance in the proposed IoT system to guarantee the integrity and availability of the video surveillance system, these characteristics are not taken into account. Besides, in Reference [5], an energy monitoring system based on IoT was proposed that uses an Android graphic interface to measure and show the excessive use of electricity in the home. In this work, the information is shared with users through an Android app; nevertheless, methodology or tools to provide security and data reliability are not provided. According to data collected by Cisco, in 2015, there were about 10 billion devices in some way connected in a network between sensors, microcontrollers, and smart homes’ sockets, among others, and it is expected that in 2020, this number will exceed 31 billion [6].

In the current context of IoT, there is a need to develop architectures that are aware of the environment where they operate (industrial, rural, or urban). In these systems, Wireless Sensor Networks (WSNs) play a leading role. In a WSN, the distributed devices use sensors to monitor different conditions, such as temperature, sound, vibration, pressure, movement, and pollutants, to name a few [7]. Sensor nodes in WSN are responsible for delivering data with a high degree of reliability, with low energy consumption, high performance, and high autonomy level. This fact can be reflected in several areas for which WSN is proposed as a solution to different needs. In this sense, the application of different security methodologies in WSN is of great interest for many researchers around the world due to the number of IoT-based applications that can be developed. For example, the author of Reference [8] proposes the use of WSN in a rural area to monitor environmental variables such as temperature, humidity, and solar radiation. In this research, devices in the network can be placed as far as 800 meters, reaching an adequate signal strength with the use of long-range (LoRa) antennas. However, only the availability of data is ensured, obtaining a 20% data loss in the transferred information. Also, security issues for data communication are not mentioned. On the other hand, a blockchain-based distributed collocation storage architecture for a WSN is proposed in Reference [9]. In this work, performance evaluation is developed by a simulation in which blockchain-based storage architecture has greater resistance to attack, high security, and is suitable for the secure storage of data transactions. Nevertheless, the authors express their need to use encryption systems for local data transmission, which cannot be applied due to the hardware topology of their proposed system. Furthermore, the authors of Reference [10] presented a cluster-tree architecture for the WSN to use in tactical military applications. In this work, organization and distribution of energy resources assure the network connectivity, low probability of interception, and detection in the cluster. In order to increase data availability, a self-organizing algorithm is proposed since the use of traditional methodologies in commercial WSNs is not recommended in military applications. Related to industry 4.0, Reference [11] shows that the use of WSN in a manufacturing line minimizes the downtime and maintenance costs. In this paper, the authors propose to deploy a sensor network system that uses built-in accelerometers to evaluate the vibrations in the manufacturing line and determine both the motion damage and component performance. The network communication employs the Message Queuing Telemetry Transport (MQTT) protocol, sending the data to a central unit where it is processed and displayed graphically to perform the corresponding procedures. However, the MQTT protocol provides only a certain degree of reliability for data and devices in the IoT systems [12]. If an unknown device in the network begins to send data to the broker, and if its topic is not registered, the message will be lost. The signal reaches the broker but will be ignored. This feature not only works to provide reliability to the data in parallel but also helps to prevent system attacks, such as a Distributed Denial of Service (DDoS) attack.

Besides, for urban environments, data integrity is an important aspect, as in the case of Reference [13], where a WSN is applied to real-time air pollution monitoring and the study of factors that influence this phenomenon. In this work, a unique identifier (id) was assigned to each WSN node. The main node verifies this id, when the data reaches the server. It is a good technique to partially ensure the integrity of the data, although the data can be modified during the transfer from the node to the central server. In this case, some cryptographic tools can help to solve this problem. Likewise, in Reference [14], a WSN-based application in the urban context is used to monitor water quality. This proposal applies a system with low energy consumption to increase its autonomy, but the fundamental aspects of security, such as integrity and data reliability, are not mentioned. According to the works mentioned above, WSNs are designed in the same way: several autonomous devices equipped with low-power microcontrollers and sensors that monitor a physical phenomenon. The main node of WSN receives and collects the transmitted data from a communication protocol and, based on the information, may or may not send a response. This generic design of WSN works optimally for general objectives. Still, it has several weaknesses in the security field, which must be improved. The lack of data integrity means that an attacker can intercept and modify the transferred information or data can be lost, as in some applications, the distances between nodes exceed the range of 500 meters. In this sense, the centralization joint with a lack of autonomy in traditional WSN is a weak point where attackers are focused on breaching the system. This dearth of security represents a vulnerability not only for the integrated elements in the WSN but also for the users who access the network. According to Reference [15], the increase in attacks on IoT systems from 2018 to 2019 was over 200%. The aggressors’ method is to attack the sum of all the vulnerabilities of the IoT system. When the attackers become familiar with the attack surface, they try to breach and take advantage of the WSN through the attack vector, which allows the attacker to use the devices for something other than their purpose [16]. WSN attacks can be classified into two types: active and passive attacks. Passive attacks are straightforward to execute and very difficult to detect [17]. Camouflage adversaries, monitoring, and eavesdropping are some examples of passive attacks.

In the active attacks, the attacker tries to remove or modify the messages that are transmitted through the network [17]. For this reason, several methodologies have been proposed to detect and prevent attacks on WSNs. For example, the authors of Reference [18] propose a robust, transparent, flexible, and energy-efficient blockchain-based authentication mechanism, specially designed for devices with restrictions on computation, storage, and power consumption. In this work, a public blockchain mechanism called Etherum is used, ensuring security, reliability, and data integrity to share information with users outside the network. During the local data transmission, a private identifier (id) is assigned to the sensor to apply a control scheme on the data origin. In Reference [19], a framework of modified blockchain models, suitable for IoT devices, was created. This framework depends only on the distributed nature and security properties of the applied blockchain. Likewise, Casado-Vara et al [20] exhibits an architecture that combines WSN and the blockchain mechanism, presenting the functionality regarding the decentralization of the system, as well as the operation of collection data, but leaving aside characteristics such as integrity, reliability, and security in data transmission and the use of encryption tools. Rose et al [21] clusters the sensor nodes and calculates the timestamp from one node to another. In this methodology, the network nodes are considered infected if the measured transmission time exceeds some threshold. While deciphering the receipt acknowledgment, this technique detects the signature mismatch and jamming in the system, stops, and creates another route for the transmission. As mentioned earlier, passive attacks are the most difficult to detect and occur predominantly in the WSN traffic. To solve this type of problem, Mehetre et al [22] presents a reliable and secure routing scheme that uses a two-tiered security mechanism and a double guarantee scheme to detect the hostile node and secure the information package in WSN. In this paper, the experiments were performed in a graph simulator, where each graph represents a node in the WSN. This means that the effectiveness of the proposal was only experienced through simulations. Another security methodology is presented in Reference [23] in order to prevent active attacks. This approach proposes a data encryption system called Talos, which can run on devices with limited energy consumption. Each of these proposals share some common elements. These approaches are aimed for IoT systems and their performance is tested in simulation software, as in Reference [24], where the goal of the presented work is to share information with users outside the local network. Although, the reported experiments used different simulation software, like Network Simulator 2 (NS2) in Reference [25], MatLab in Reference [26], and Cooja simulator and Network Simulator 3 (NS3) in Reference [27], to name a few. Until now, we talked about conceptual proposals, as in Reference [28], where a blockchain model was proposed for access control, integrity, and data reliability. This work applies a robust private blockchain design but does not take into account the computational cost of the devices in the system. Due to this, the proposed work in Reference [28] cannot be implemented in some device architectures. This happens because the elements, used in the WSN design, have normally low computational and resource consumption, as in References [29,30,31], which are regularly used elements in this type of IoT infrastructure.

On the other hand, in traditional WSN, which implements robust encryption methods to solve computational cost, these problems become a problem in the performance and functionality of the IoT infrastructure system. There were mostly theoretical works on the combination of WSN and blockchain; therefore, it would be convenient to have a blockchain-based methodology for transferring the collected information from the IoT devices to the computing cloud to share it between different users. Furthermore, in a centralized WSN architecture, the task of sharing information between different users is not only local but also external to the information network. These issues cause data integrity and availability problems because they can be easily modified. In this work, a symmetric encryption method is proposed to increase the integrity and confidentiality of the data travelling over the network. The proposed methodology is carried out through the transmission of data between physical devices and the main data receiver. This decentralized infrastructure is implemented in an IoT system, a wireless sensor network with a publish-subscribe architecture, which uses a light communication protocol between devices.

## 2. Theoretical Background

The Bitcoin cryptocurrency was proposed by Satoshi Nakamoto in Reference [32]. This work defines a cryptocurrency as a medium of exchange as traditional currency, like the Dollar. The cryptocurrency is designed to perform the exchange using cryptography principles [33]. In other words, Bitcoin is the digital token, and blockchain is the realized methodology to track all transactions and operations of the digital tokens [34]. Bitcoin cannot exist without blockchain, but there can be blockchain without Bitcoin [35]. That means that blockchain is not constrained to the financial area, which allows recording any information shared on the internet through blockchain [36]. Daily, large amounts of cryptocurrency transactions occur around the world.

**Figure 1:**
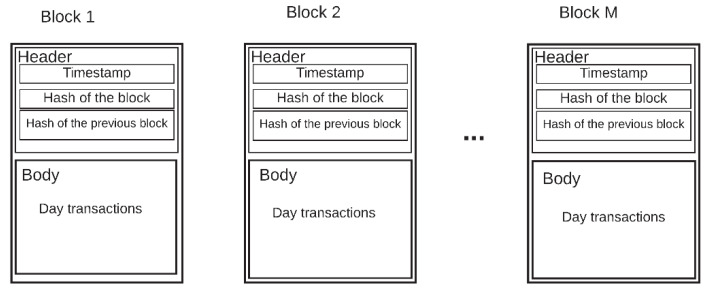
Blockchain general structure.

Each record includes specific information, such as payer, beneficiary, time, date of the transaction, and may have additional information if required [36]. These datasets are called blocks. The blocks are uploaded to the internet in chronological order due to each block being a set of data. These blocks can be manipulated and falsified independently in an effortless way. To stop this, a hash function can be used, such as Secure Hash Algorithm (SHA)-256, developed by the National Institute of Standards and Technology, USA (NIST) [37]. The hash function is an irreversible calculation protocol, where a hash value can be calculated from the source data, but not vice versa. When a new block is added to the chain, the hash is calculated from the new block, as was done with the previous block. The hash is registered in the new block, along with the information it contains. If someone wants to make a modification or forgery in the block that has just been added, the discrepancy between the data and the hash is detectable [38]. If the new block was independent, no one would notice the discrepancy, but as its hash is calculated using the hash of the previous block, the discrepancy is notorious. To fix this, all the hashes of all the blocks must be falsified, and this task is very exhaustive, which requires too much effort, and that is why the blockchain becomes immune to counterfeiting. Figure 1 shows the general structure of a blockchain. The header contains the hash value of the current block, the previous block, and a timestamp. The body part consists of transactions or data.

There are three types of blockchain: public, private, and hybrid [39]. The public type implies that the blockchain model is public and data is open, and anyone can consult, validate, and make use of the model. Some examples are Bitcoin, Ethereum, Monero, Dash, and Litecoi. The private blockchain is the opposite and it depends on the policies of the organization that allows access to the company or network. Finally, there are the consortium or permit networks, which are hybrid models between public and private [40]. In IoT systems, blockchain is applied like a communication protocol between devices, as in Reference [41], where a comparison between a communication protocol MQTT with blockchain is carried out. In the obtained results, the authors express how the use of a public blockchain model in an IoT system helps to solve security problems. However, this work only uses robust resources due to the public blockchain mechanism that is used. Another way to implement the blockchain in IoT systems is presented in Reference [42]. In this work, the use of blockchain to generate blocks of all collected information from an IoT system is carried out. This fact represents a more optimal way of embedded IoT systems. A detail with this proposal is that when data is collected and travels to the storage, there is a vulnerable point in the system.

**Figure 2:**
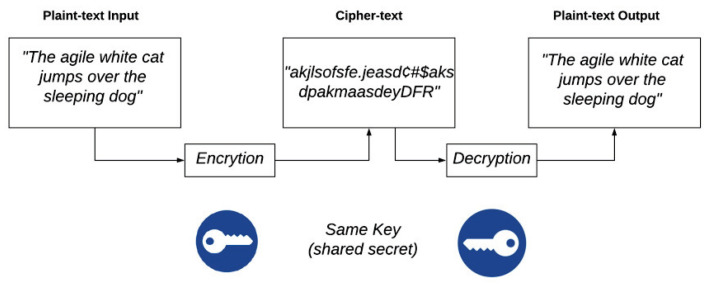
Operation scheme of Advanced Encryption Standard (AES) encryption algorithm.

Several authors propose different solutions for this detail with the use of cryptographic algorithms [43]. A cryptographic algorithm modifies the data in a document to achieve some security features such as authentication, integrity, and confidentiality [44]. An example is the Advanced Encryption Standard (AES). AES is a symmetric encryption algorithm with a high degree of security. Figure 2 shows the AES encryption process. Encryption converts data into an unintelligible form called encrypted text, while decryption converts this encrypted text into original plain text. The AES algorithm can use 128-, 192-, or 256-bit cryptographic keys to encrypt and decrypt data in 128-bit blocks [43]. Other way to secure the blockchain is to use authentication tools like in [45], where the authors propose a new light authentication called LBRAPS based on bitwise exclusive-or (XOR).

## 3. Methodology

**Figure 3:**
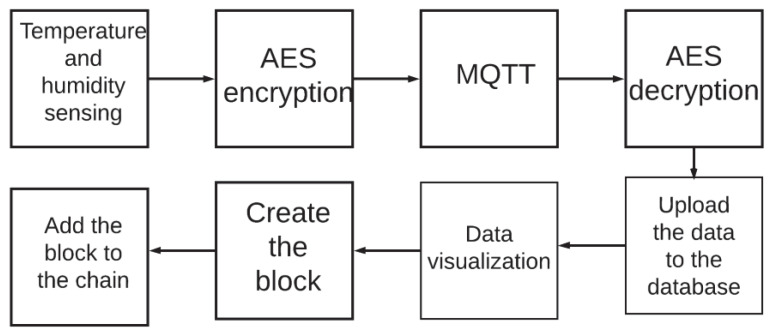
Proposed methodology.

Figure 3 shows the proposed methodology integrating blockchain and symmetric encryption in a wireless sensor network system to monitor physical variables like temperature and humidity. 

First, the sensors measure the temperature and humidity. The wireless sensor network was deployed in an office at the university’s faculty of engineering, where usually between three to ten people work. Temperature and humidity were measured every minute. The temperature inside the office ranged between 19 and 21 °C. Once obtained from the sensors, the data was encrypted with the AES methodology. The private key was statically assigned based on two parameters: the number of busy nodes in the WSN and the signal type used for the temperature or humidity monitoring. At this point, the encrypted data travels to the receiver using the MQTT protocol, which applies a topic-based publish-subscribe architecture. These topics are the monitored variables, such as humidity and temperature. Once the message reaches the recipient, the data is decrypted with the private key that was used for the encryption. After the information goes through the decryption process, it uploads the information to a local database along with the timestamp from when the information was entered and the node ID of where it came from. In parallel, the data can be visualized to interpret and observe the signals. When there is a considerable amount of data in the local database, the blockchain block is created with all the records that are in the database. The block is added to the data, along with the rest of the blocks containing previous records of the WSN. This is how the chain is formed into a blockchain, sharing the data with different users, and in the meantime, ensuring that modifications in the data or changes in the chain do not occur. 

### 3.1. Performance Indices

#### 3.1.1. Key Characteristics of Blockchain 

The blockchain as a new module contributes to the security of the WSN with its characteristics. To have data integrity and traceability in a blockchain, the authors of Reference [46] concluded four attributes from other research. From these, we considered three to be relevant in a WSN:

- Autonomous: This is considered one of the most important aspects of a blockchain system, as no one is controlling or governing the system.
- Immutability: Blockchain is used for security purposes in many applications because it cannot be manipulated without a trace using currently known technology.
- Contractual: The blockchain creates consensus between the chains or peers. The consensus process is executed autonomously using rules according to the data status, contributing to the full autonomy of the system.

These characteristics are essential in various fields of the Internet of Things. The authors of Reference [47] described a Cloud-Based IoT network architecture, with a need for system autonomy and incorruptible data storing blocks. Also, a blockchain system for industry 4.0 was presented in Reference [48], and the study agrees on how decentralization, verifiability, and immutability can be used to improve security (along with other cryptographic tools).

#### 3.1.2. Data Security in Wireless Networks

Providing security in wireless sensor networks differs from traditional approaches due to resource limitations and computational restrictions. A sensor network can be called secure if it can provide end-to-end security, fulfilling the requirements of confidentiality, authenticity, and data availability [49]. The sheer number of interconnections between thousands of sensor nodes in large sensor networks can cause technical issues such as interoperability issues, lack of service quality, and data accessibility problems. To evaluate the security systems for existing wireless sensor networks, the authors of Reference [50] propose to use requirements such as data confidentiality, authenticity, and availability. Also, they mention how cryptographic and security authentication tools like key management, symmetric, asymmetric, or hybrid encryption provide various security options for the sensor network. In Reference [51], evaluation points for wireless sensor network security are proposed. The first four points have primary importance, and the last is important in some sensor network systems.

1. Data availability refers to ensuring robust security in the sensor network, to protect the resources or the sensor nodes. The nodes in the sensor networks must have self-protection to avoid unnecessary message processing or task execution. This can reduce energy use and increase the life span of the sensor network. Wireless sensor networks are susceptible to multiple types of denial of service attacks, node compromise attacks, and resource consumption attacks [52]. Therefore, the availability and security of resources must be effectively maintained.
2. Data confidentiality can be achieved by allowing only authenticated users to access data or devices in the system to send messages. In sensor networks, data can be protected using cryptographic methods to allow only authenticated users to access it. Unauthorized or third parties cannot read the original data if confidentiality is effectively provided [53]. Therefore, to have data confidentiality, wireless sensor networks must use encryption methods.
3. Data authentication in sensor networks allows the system to verify whether data is sent from authorized sources or not, and also protects the original data from alterations.
4. Data integrity in sensor networks is necessary to verify the information reliability and capacity, to ensure that the message has not been damaged, altered, or changed. The integrity of the network is violated when the malicious node in the network sends false data with a valid identifier id.
5. Time synchronization is important in systems with sensor node uptime coordination, as a more collaborative sensor network may involve group synchronization.

These security assessment features for wireless sensor networks are applied in various fields of IoT. Reference [54], References [55] and [56] in Medicine 4.0, Reference [57] in mobile cloud computing, and Reference [48] in Industry 4.0 agree that data availability, confidentiality, authentication, and integrity are evaluation points in security systems which must be complied with in the implementation of IoT. As each field uses a different variation of information from multiple types of users that use a different kind of technology, these general security requirements must be set as security goals. 

#### 3.1.3. System Performance Metrics

The exponential growth of development in low-power electronics, ubiquitous smart sensors, and Wireless Sensor Networks (WSN) produces a wide range of monitoring and tracking applications. These technologies must meet with various quality requirements. The authors of Reference [58] proposed evaluation metrics for Quality of Service (QoS). 

Latency is the delay experienced by packet during the travel from the source to the receiver node. The network layer can achieve minimal latency or end-to-end delay by calculating the shortest route or the route with the least obstruction for the packet. According to Reference [59], the Message Queue Telemetry Transport (MQTT) communication protocol counts with a latency of 40 ms. This is considered a low time compared to Reference [60], as other protocols can reach 1000 ms in latency. On the other hand, even though the use of blockchain could affect the latency of the system in general terms, it can still cope with an even higher level of delays. According to Reference [61], several authors implemented Etherum, a public blockchain model, causing 15,000 ms of lag, and their IoT systems were able to handle it. While using private blockchain designs, they experienced only 8000 ms latency [62]. 

Reliability is the network’s ability to transmit information in real-time. To ensure this, a light communication protocol can be used, or if the system has to cover large areas, several receiver points can be placed. The studies of References [63,64,65] mention that the lower the latency, the greater the reliability.

The throughput is the number of packets arriving safely at the system per second. In the work of Reference [66], their 3-node system presents an interval of 2.12 to 2.81 messages per second, with generic microcontrollers such as ESP8082 and Arduino Yun Mini. 

Adaptivity or scalability is the system’s capacity to adapt to the changes, like adding or removing nodes from the network or any alteration that could affect the stability of the systems. 

In addition to these characteristics, the Central Processing Unit (CPU) frequency, the percentage of CPU usage, and the system temperature are important to quantify the system’s performance and lifetime. The authors of Reference [67] mention how the important factors are the CPU frequency and the percentage of the CPU, as adequate use of these two considerably lengthens the lifetime of the system. Likewise, the authors of Reference [68] mention the importance of the system temperature. If it exceeds the limits, the system can be irreversibly damaged, or it can simply hinder its performance, affecting the variables such as adaptivity or reliability, as well as the security and integrity of the data.

The selection of these characteristics to measure the performance of an IoT system agrees with the works of References [54,55,56,57,69,70,71]. They agree that low latency is an important characteristic of IoT systems. According to the authors of Reference [54], the communication protocol plays a major role in this property and low latency helps the interoperability of the system. The authors of Reference [56] argue that scalability and reliability are also important as the size of the IoT systems are constantly growing.

These performance characteristics in wireless sensor networks can be affected by the computational complexity of some encryption tools. According to the authors of Reference [72], the computational cost of the encryption algorithms depends on the number of encryption cycles or rounds, which is dependent on the size of the key that is used for the encryption. Table 1 presents a comparison of six encryption tools with different key sizes, block sizes, and numbers of rounds.

According to the authors of Reference [73], cryptographic tools with more than 16 rounds tend to be more robust, but it is not recommended to use them in WSNs directly because of their slow performance and exceeding memory requirement. The results reported in Reference [74] show that encryption algorithms which use 64-bit keys to preserve the privacy of your information can be broken in 3.5 months with supercomputers which test 1012 passwords in a second. While encryption algorithms using 128-bit keys at this time are valued at 5.4 × 1018 years. According to the authors of Reference [73], the security solution developed for wireless sensor networks must be modular. The number of rounds in the AES encryption algorithm depends on the size of the encryption key. If the size of the key is 128, the system uses 10 rounds, if it is 192, it will use 12, and if the key size is 256, the number of rounds is 14. This method does not require complex computing and energy-intensive procedures and works simply by dividing a message into 128-bit blocks in length and sequentially encrypting its 128-bit key. This algorithm has been tested in the ZigBee modules of practically all modern manufacturers, finding a balance between security and system performance [75].

## 4. System Architecture

**Figure 4:**
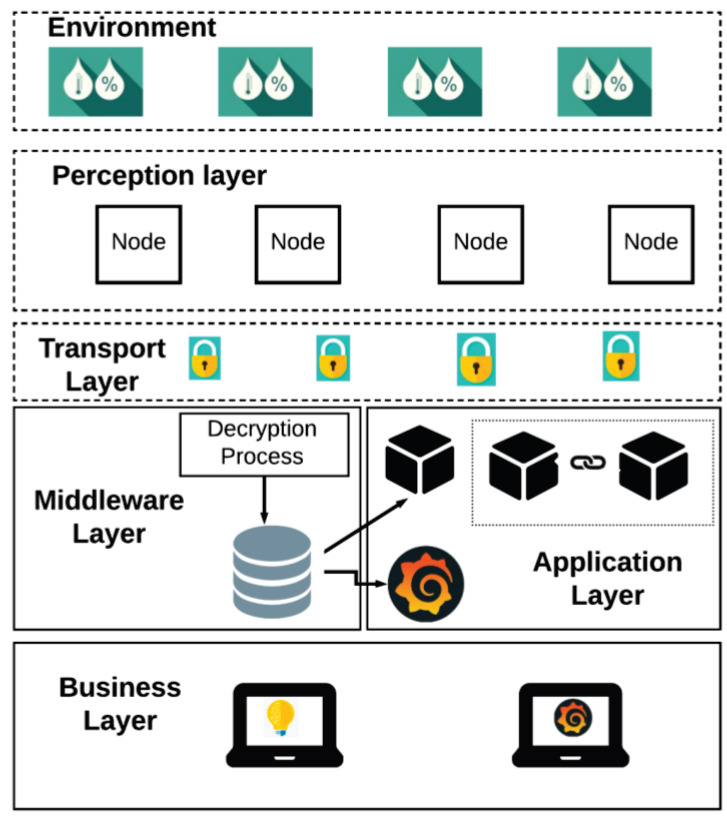
Architecture system scheme of Wireless Sensor Networks (WSN) together with the blockchain mechanism and symmetric encryption.

The proposed system is designed according to the layered architecture of an IoT system and it is outlined in Figure 4. The first layer represents the environment with the monitored signals such as temperature and humidity. The second is the perception layer, where the WSN is located with three sensor nodes, each consisting of a Telaire T9602 sensor and a Pycom brand WiPy 3.0 microcontroller. The microcontroller works with python programming language version 2.7 with access to a great number of libraries and applications. In the transport layer, the encrypted data travels to the receiver using the MQTT protocol. This protocol was selected for its favorable characteristics like scalability, the secure message sending and receiving, minimum bandwidth, energy consumption and processing, and its publisher/subscriber architecture, among others. The middleware layer is essential not only for the proposed methodology but also for the MQTT protocol and the publish/subscribe architecture based on topics. For the development of the IoT system of WSNs, a Raspberry Pi 3B+ model was used. This layer has the function of being an intermediary of messages in the wireless sensor network. In parallel, it decrypts messages with the AES methodology and moves the records to the database.

The application layer is where the blockchain and the "Grafana" graphic display system are located. The visualization of the data is in real-time while the page is being updated. In the proposed embedded system, the blocks are generated with all the records contained in the database, and each block is uploaded to be part of the blockchain. In the last section, we can find the end-users who access the data manually in the blockchain through an administrator for different purposes. After accessing the data, the users can work with the data but cannot modify it. If there is a modification, the chain is broken, and the blockchain system is affected.

**Figure 5:**
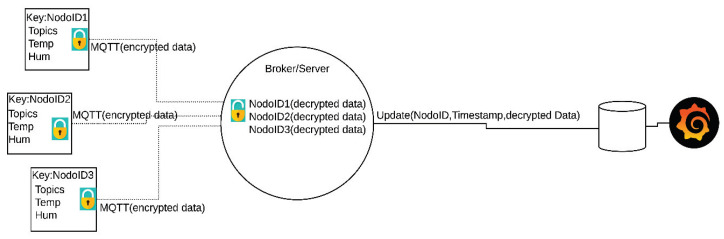
Data encryption operating scheme with the Message Queue Telemetry Transport (MQTT) communication protocol.

The operation of symmetric AES encryption in conjunction with the MQTT protocol is described in Figure 5. This figure displays how the data travels encrypted through the network with the MQTT protocol. The encryption key in this example is the node ID with a size of 128-, 192-, or 256-bits. For security reasons, only the node and the broker know the key. 

**Figure 6:**
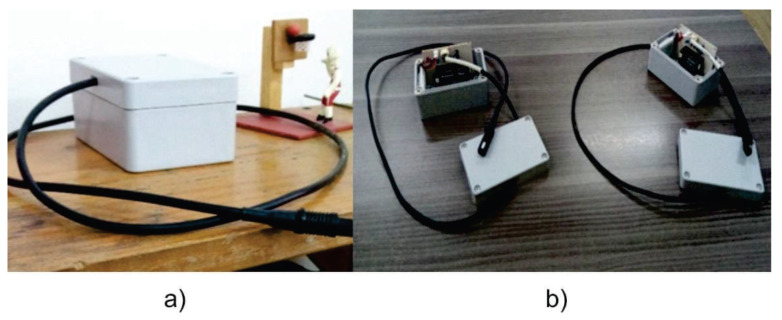
(**a**) Wireless sensor node monitoring environmental data, (**b**) wireless sensor nodes and their internal structure.

**Figure 7:**
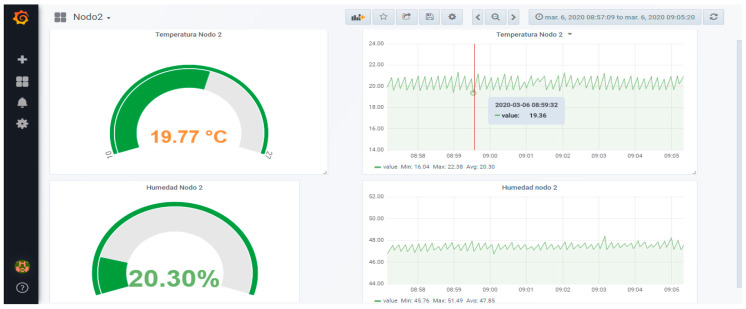
Graphic display of information collected by the sensor network system.

Figure 6a represents how one of the sensor nodes works during the test. Also, Figure 6b shows the internal structure of the node, the Wipy 3.0, and the Telaire T9602 humidity and temperature sensor. On the other hand, Figure 7 presents a screenshot of the Grafana data visualization interface that displays the information from the database in real-time.

### Blockchain Design

**Figure 8:**
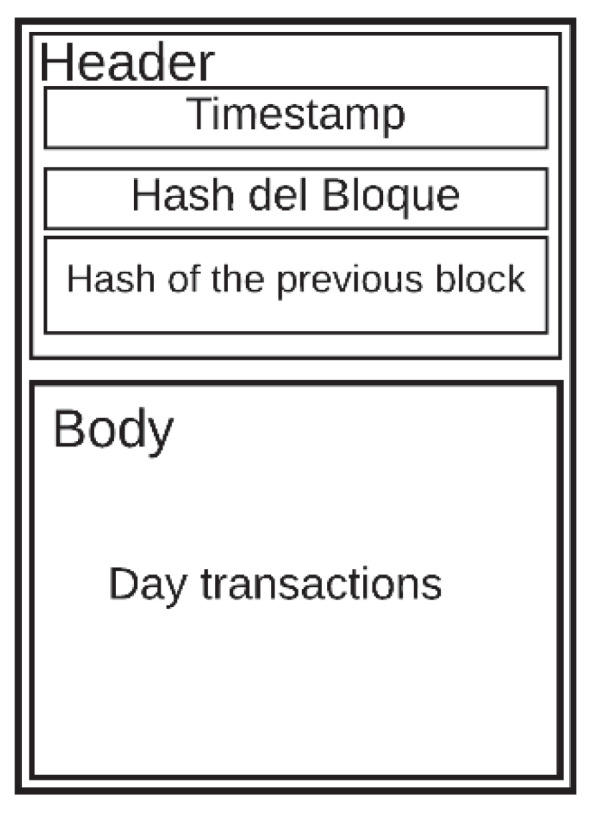
Blockchain design.

The transaction or block in the chain (Figure 8) has two parts: a header and a body. The header has four attributes:

- Timestamp: This attribute is necessary to know when the block was created in all transactions.
- Block hash: This hash is important because it will be linked to the future block. The hash is created based on the transactions of the day. If a piece of information is modified, the hash no longer matches the one registered in this field.
- Block number: This data represents the position the block occupies in the chain to know the number of blocks that the blockchain system contains.

The body contains all the records or transactions of the data in the database that were made during the day or at a certain time. 

**Figure 9:**
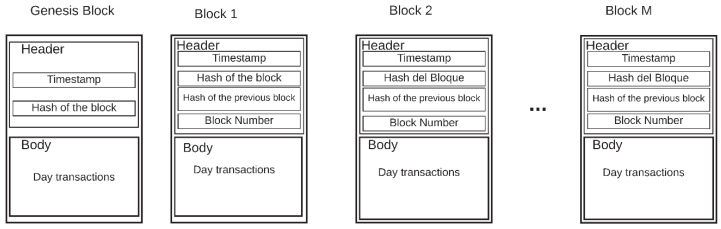
Composition diagram of blockchain system blocks.

Figure 9 exhibits the creation of the blocks and their unification in a blockchain system. The first block is the genesis block. In the original blockchain design for Bitcoin, Satoshi Nakamoto proposes this block to be the basis of the construction of the entire chain [32]. The design of this block contains all the fields except the hash of the previous block.

## 5. Results

Hardware performance was tested in two scenarios. First, the sensor network system operated with the symmetric encryption mechanism and the proposed private blockchain system. In the second scenario, the sensor network system without the proposed safety mechanisms was tested to show that the methodology can be implemented in a low-resource architecture. In the test scenario, a Raspberry Pi 3B+ was used with the following architecture:

- 1.4 GHz 64-bit quad-core processor
- Dual-band wireless Local Area Network (LAN)
- 4 GB de Random Access Memory (RAM)
- Operating System (OS) Raspbian

The duration of the test in both scenarios was 120 minutes. During this time, we recorded the state of the hardware attributes every minute, such as the system temperature, the CPU’s working frequency, and the percentage of CPU usage.

**Figure 10:**
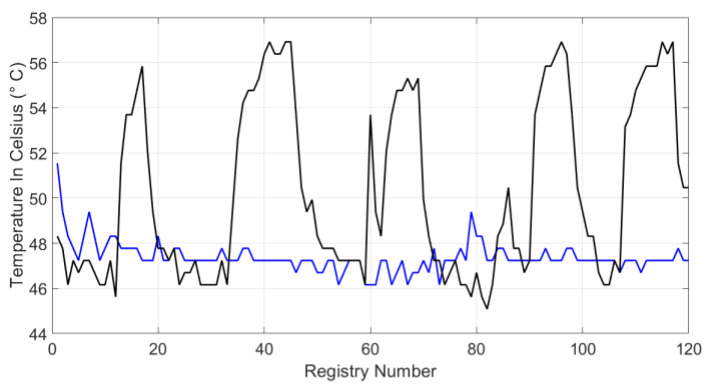
Hardware system temperature for test I.

**Figure 11:**
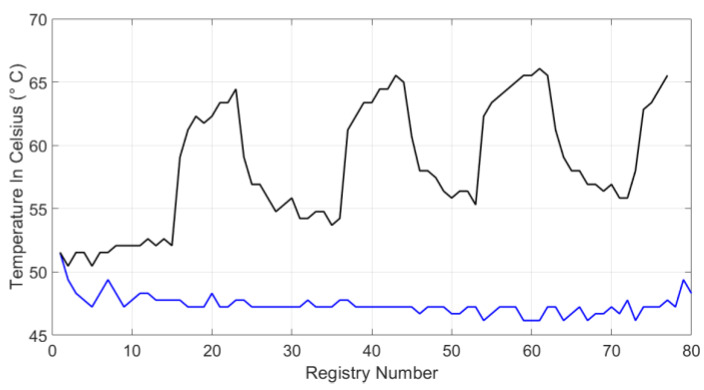
Hardware system temperature for test 2.

Figure 10 and Figure 11 show the system temperature on the Celsius scale. The black line represents the system behavior when both blockchain and symmetric encryption systems are jointly working. The blue lines in Figure 10 and Figure 11 visualize the system temperature in the second scenario, without applying the proposal. There are sections in Figure 10 over the black line, where the temperature reaches 57 °C; during these peak intervals, the chain is formed from the blocks by the blockchain system. In both waveforms, the temperature reaches between 49 to 51 °C because the graphic visualization of the data or any query in the database was performed. In Figure 11, temperature trend is shown to be maintained, however, this performance has slightly increased as the system was being used for other functions, such as data consultation and graphical monitoring.

**Figure 12:**
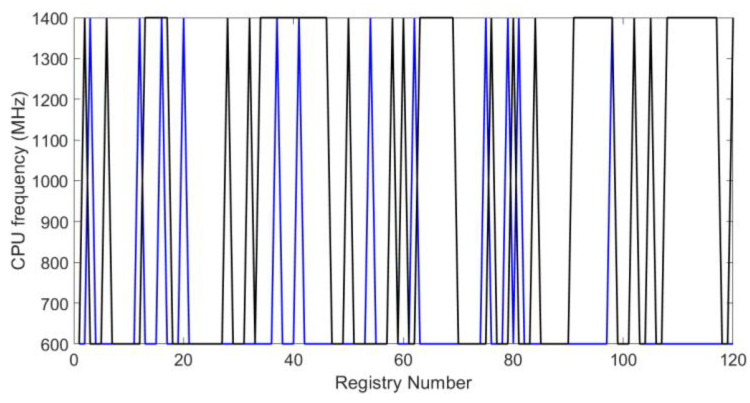
CPU usage frequency for test I.

**Figure 13:**
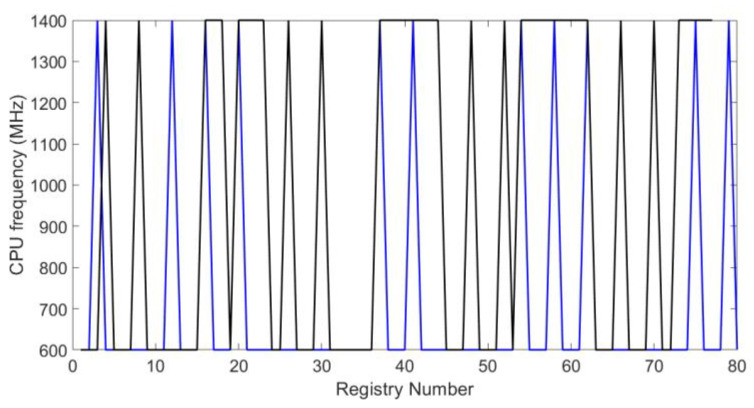
CPU usage frequency for test II.

The graphs of Figure 12 and Figure 13 show the CPU frequency of the embedded system. In the Raspberry Pi 3B+, frequency ranges from 600 to 1400 MHz. In this figure, the black line represents the system behavior when both blockchain and the symmetric encryption system operate. In the same way, the blue line represents the behavior without the proposed methodology. These waveforms show intervals where CPU frequency is extended to a total of 1400 MHz. This happened when the blockchain block was created for the IoT system and this was uploaded to the data cloud.

**Figure 14:**
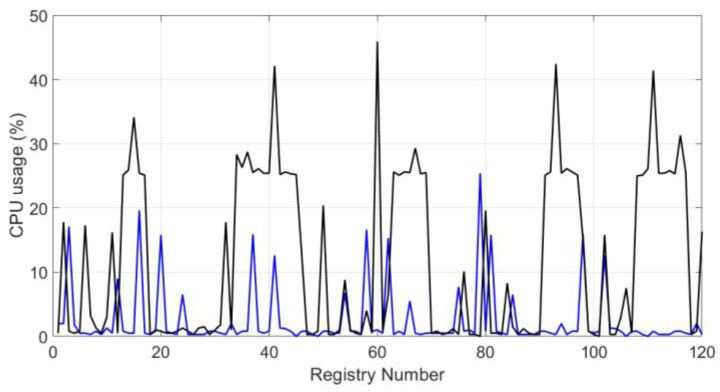
CPU usage percentage for test I.

**Figure 15:**
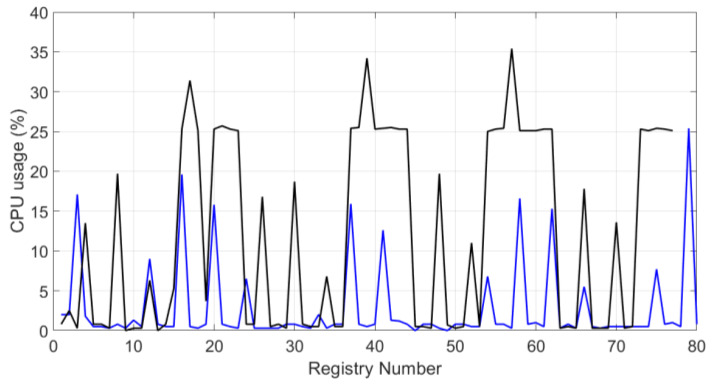
CPU usage percentage for test II.

Besides, Figure 14 and Figure 15 represent the waveforms of the system CPU usage. The black line represents the behavior of the system when the blockchain and the symmetric encryption system are running, and the blue line represents the behavior without the system. In the Raspberry Pi embedded system, the maximum percentage is 100%. These graphs show how the system does not use 50% of the capacity in both study cases. 

## 6. Discussion

### 6.1. Hardware Performance

Based on the results of the previously presented experiments, we can identify several vital points in the proposed wireless sensor network architecture. We can observe in the graphs of the temperature and the percentage of CPU usage that not all resources are used. Scalability is one of the essential features in wireless sensor networks and any IoT system. During the tests, we observed that adding or removing nodes does not disturb any aspect of the architecture or methodology, the blockchain system and symmetric encryption continue to work without any problem or delay in sending packages. 

According to Reference [76], the temperature limit on a Raspberry Pi 3B+ is 85 ° C. If this limit is exceeded, the embedded system suffers damage in its structure and operation. In both testing scenarios, we noted that the temperature never exceeded 60 °C. Furthermore, according to Reference [41], the system could present problems in its performance and the interoperability of the entire infrastructure when the CPU usage reaches 90% or 100%. The obtained CPU usage results (Figure 14 and Figure 15), applying the proposed methodological approach, remain below 50% of the capacity of those in Reference [42], and occupy between 98% and 100% of all the capacity of the systems. It can be noted that despite using a robust architecture, their proposal turns out to be too heavy to be implemented in a low-resource system. 

The system’s performance analysis, using the metrics defined in Section 3.1, shows that the presented proposal has a good balance between security and resource consumption. Features such as low latency and reliability were achieved thanks to the MQTT protocol. The latency of 40 ms can be considered an acceptable value, compared to other protocols where latency can go up to 120 or 1000 ms. The reliability is reflected in the number of packets connecting to the system per second, which was 1.94 packets per second in a space of two hours of experimentation. The graphs of the system performance (Figure 10, Figure 11, Figure 12, Figure 13, Figure 14 and Figure 15) show that the low-resource usage was not altered by the use of cryptographic tools, thanks to the low computational consumption of the AES. Also, despite the low-power usage, AES provides a high degree of security, reliability, and integrity to the data transmitted over the network. Adaptivity or scalability is possible due to the low consumption of resources. Adding or removing an N number of nodes does not affect the interoperability of the system. Neither the security system nor any level of infrastructure was affected, as the graphs of system performance (Figure 10, Figure 11, Figure 12, Figure 13, Figure 14 and Figure 15) show. It is demonstrated that the use of cryptographic tools of average computational usage, such as AES, has a significant impact on the system to have a good performance, provide security, reliability, integrity, and availability to the system, and also do not interfere with the interoperability of infrastructure.

### 6.2. Security Analysis

Table 2 presents a comparison of two investigations against our proposal. These works were selected based on the following criteria: (1) To be set in a real scenario, and (2) to address the same research topic as this work, a security proposal for IoT systems which has been implemented in a real infrastructure without simulations. The evaluated performance indexes are:

1. Basic security: As stated in Reference [77], it is defined in three fundamental security aspects: confidentiality, integrity, and availability (CIA).
2. DDoS attack: It is understood as the resistance or not to this type of attack, based on its architecture and model.
3. Linking attack: It is identified as the resistant or not to this type of attack, based on the architecture it proposes.
4. Interoperability: This feature refers to whether all the model layers exchange information and use the exchanged information.
5. Cryptographic tools: Tools that were used or not used for the development of the IoT system.
6. Type of blockchain: Identified as private, public, or consortium.

The comparison between the models of Reference [77,78] with our proposal makes evident some differences. In our project, Hashwas used as a cryptographic tool in the blockchain during transactions with different organizations. Besides, the AES tool was used in the data transport layer. There are numerous advantages to using these encryption tools. HASH maps arbitrary size data to a fixed size string. AES is a symmetric encryption tool that requires a private key for encryption and decryption of information, the length of which must be 128-, 192-, or 256-bits. Furthermore, they are designed to be a one-way function. So, the only way to get the input data from the HASH code is by brute-force searching for the possible inputs or using a table of matching hashes. Moreover, the proposed methodology applies private blockchain while the other methods used public blockchain for their model. This choice makes the proposed model more robust, and the design adaptable to other needs of the sensor network system, adding more features as the IoT system grows. Table 3 presents the characteristics, pros, and cons of each related scheme.

## 7. Conclusions

Risk growth and analysis are among the main concerns in the development of the IoT paradigm. This research presents a decentralized infrastructure for IoT systems, which guarantees the security, privacy, reliability, and autonomy of the system, and where smart devices can securely communicate with each other. This proposal of a decentralized approach can be implemented in most IoT infrastructures, different contexts, and services. It is based on a private blockchain mechanism; therefore, it benefits from its security properties. Also, we analyzed the computational consumption of infrastructure and data security. The analysis was performed based on performance indices widely used and mentioned in various investigations and implementations in the field of IoT, industry 4.0, medicine 4.0, and cloud computing, among other fields. Based on these evaluations, we can conclude that the proposed methodology has a low-resource consumption and fulfils the fundamental requirements of security, confidentiality, authenticity, and availability. Besides, the security analysis shows that the decentralized infrastructure is less susceptible to the most common attacks on IoT systems, such as DDoS, man-in-the-middle, and linking attack. On the other hand, the interoperability and autonomy of the system are not affected by adding or removing smart devices from the infrastructure, and latency in the network is kept low by the proposed communication protocol. Another point to conclude is that based on the performance indices, the security of the presented proposal can be implemented in various IoT contexts, such as industry 4.0, medicine 4.0, and in smart homes, among other fields.

## Figures and Tables

**Table 1:** Comparison of investigations with the contribution in this paper.

Cryptographic Tool	Key Size (bit)	Block Size (bit)	Round
*DES	56	64	16
*3DES	168,112,64	64	48
*DES-X	184	64	16
*AES	128,192,256	128	It depends on the block size
**Skipjack**	80	64	32
*HIGHT	128	64	32

* Data Encryption Standard (DES), Triple Data Encryption Algorithm (3DES), Data Encryption Standard-X(DES-X), Advanced Encryption Standard (AES), HIGh security and light weigHT (HIGHT).

**Table 2:** Comparison of investigations with the contribution in this paper.

Security Criteria	Reference [78]	Reference [79]	Current
**Basic Security Aspects**	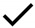	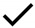	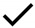
*DDoS attack	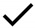	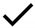	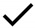
**Linking attack**	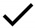	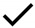	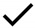
**Interoperability**	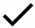	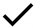	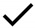
**Cryptographic tools**	---	---	Hashand AES
**Blockchain type**	Public	Public	Private

*Distributed Denial of Service (DDoS).

**Table 3:** Characteristics, pros, and cons of each related scheme.

	Advantages	Disadvantages
**Basic Security Aspects**	Confidentiality, integrity, and availability to the IoT system and the information it shares.	It can disturb the performance of the system, it depends on the resources that are used.
**Decentralization**	The decentralized approach helps the overall security and integrity of the system.	It could represent a high computational cost for the system.
**Topic-based publish-subscribe architecture**	Prevent unknown devices from spamming the system, trying to spread malware, or launching a Distributed Denial of Service (DDoS) attack.	A static architecture could be represented as a limitation for some IoT systems.
**MQTT communication protocol**	Due to its characteristics, it can boast of having a really low consumption as well as using very few resources for its operation.	Being designed as a lightweight protocol, the use of cryptographic tools for data transmission is not contemplated.
**Cryptographic tools**	Integrity and reliability of the information that is shared locally and externally.	It could represent a high computational cost for the system, depending on the resources used and the way they are applied.
**Blockchain type**	A private blockchain is custom-designed based on the needs of the system.	The needs of the IoT system change according to time and interaction with users. Blockchain’s designs, being more robust, cover the needs of the system without having to make changes to the design.
